# Proteomic Profiling and Epitope Analysis of the Complex α-, γ-, and ω-Gliadin Families in a Commercial Bread Wheat

**DOI:** 10.3389/fpls.2018.00818

**Published:** 2018-06-19

**Authors:** Kyoungwon Cho, Hye-Rang Beom, You-Ran Jang, Susan B. Altenbach, William H. Vensel, Annamaria Simon-Buss, Sun-Hyung Lim, Min G. Kim, Jong-Yeol Lee

**Affiliations:** ^1^National Institute of Agricultural Sciences, Rural Development Administration, Jeonju, South Korea; ^2^Western Regional Research Center, United States Department of Agriculture, Agricultural Research Service, Albany, CA, United States; ^3^College of Pharmacy and Research Institute of Pharmaceutical Science, Plant Molecular Biology and Biotechnology Research Center, Gyeongsang National University, Jinju, South Korea

**Keywords:** gliadins, proteomic profiling, epitope analysis, celiac disease, food allergy

## Abstract

Wheat gliadins are a complex group of proteins that contribute to the functional properties of wheat flour doughs and contain epitopes that are relevant for celiac disease (CD) and wheat-dependent exercise-induced anaphylaxis (WDEIA). In this study, we extracted ethanol-soluble gliadin fractions from flour of the Korean bread wheat cultivar Keumkang. Proteins were separated by 2-dimensional gel electrophoresis (2-DE) using a pI range of 6–11 in the first dimension and subjected to tandem mass spectrometry. α-, γ-, and ω-gliadins were identified as the predominant proteins in 31, 28, and one 2-DE spot, respectively. An additional six ω-gliadins were identified in a separate experiment in which a pI range of 3–11 was used for protein separation. We analyzed the composition of CD- and WDEIA-relevant epitopes in the gliadin sequences from Keumkang flour, demonstrating the immunogenic potential of this cultivar. Detailed knowledge about the complement of gliadins accumulated in Keumkang flour provides the background necessary to devise either breeding or biotechnology strategies to improve the functional properties and reduce the adverse health effects of the flour.

## Introduction

Wheat (*Triticum aestivum* L.) is cultivated in more than 100 countries throughout the world and is a major source of protein for human nutrition. Wheat flour is used in a wide range of food products that are characteristic of particular regions and/or cultures and include different types of baked, steamed, and flat breads, tortillas, pastas, noodles, couscous, and baked goods. The diversity of products that can be made from wheat flour is possible because of the unique viscoelastic properties conferred by the gluten proteins, a complex group of 50–100 proline- and glutamine-rich proteins accumulated in the wheat grain (Delcour et al., 2012). These proteins contain regions of very repetitive sequences and are difficult to separate, identify, and quantify. The gluten proteins consist of two groups of proteins, the polymeric glutenins that contribute elasticity to wheat flour dough and the monomeric gliadins that confer extensibility to dough. The glutenins consist of high molecular weight-glutenin subunits (HMW-GS) of 70–90 kDa and low molecular weight-glutenin subunits (LMW-GS) of 30–45 kDa that are linked together by intermolecular disulfide bonds while the gliadins are divided into three major groups on the basis of structure, referred to as α-, γ-, and ω-gliadins (Payne, 1987). The complement of proteins within each group and the relative proportions of the individual proteins are important for determining the quality of the flour and its suitability for use in different products.

In addition to their role in flour functionality, some gluten proteins also trigger human health problems, including celiac disease (CD), and food allergies (FA) (Matsuo et al., 2004, 2005; Sollid et al., 2012). CD is a complex autoimmune disease that is triggered in genetically susceptible individuals by the ingestion of the wheat gluten proteins and similar proteins from barley and rye. Epitopes that stimulate T-cells of celiac patients have been identified in all classes of gluten proteins although the gliadins are thought to be the most important. Sollid et al. (2012) recently summarized epitopes relevant for CD and reported five core sequences from α-gliadins, eight from γ-gliadins, two from ω-gliadins, two from LMW-GS and one from a HMW-GS. Wheat-dependent exercise-induced anaphylaxis (WDEIA) is a serious food allergy that occurs in sensitized individuals when the ingestion of wheat is followed by physical exercise. A number of epitopes that trigger WDEIA have been identified in ω5-gliadins, a subset of ω-gliadins (Matsuo et al., 2004, 2005).

Studies on the wheat gluten proteins, particularly the gliadins, are complicated by the large sizes of the gene families, the high proportions of pseudogenes in each family, the similar and repetitive sequences of the members and the allelic diversity among cultivars. The γ- and ω-gliadins are encoded by genes at the *Gli-1* loci on the short arms of the group 1 chromosomes, respectively, and are tightly linked to the LMW-GS genes, whereas α-gliadins are encoded by genes at the *Gli-2* loci on the short arms of the group 6 chromosomes (Rasheed et al., 2014). Based on hybridization analyses, it has been estimated that a single hexaploid wheat contains from 25 to 150 α-gliadin genes, 17 to 39 γ-gliadin genes, and 15 to 18 ω-gliadin genes (Sabelli and Shewry, 1991; Anderson and Greene, 1997). Alpha-gliadins consist of one N-terminal repetitive domain, two non-repetitive domains and two polyglutamine domains. Six cysteine residues are present in the non-repetitive domains of most α-gliadins and form intramolecular disulfide bonds (Anderson and Greene, 1997; van Herpen et al., 2006). Gamma-gliadins are composed of five domains (I–V), including one N-terminal domain, two non-repetitive domains (III and V), a repetitive domain (II), and a polyglutamine region (IV). Domain II is proline and glutamine-rich and contains the basic repeat motif [PFPQ_1-2_(PQQ)_1-2_] (Anderson et al., 2013). Eight cysteine residues in the non-repetitive domains (III and V) of most γ-gliadins form intramolecular disulfide bonds. Omega-gliadins consist of short N- and C-terminal domains (I and III) and a repetitive domain (II). Cysteine residues are not observed in most ω-gliadins. The ω-gliadins are subdivided into ω1,2-gliadins (∼46–58 kDa) with N-terminal sequences of ARE-, ARQ- or KEL- and PQQPFP repetitive motifs and ω5-gliadins (∼55–65 kDa) with N-terminal sequences of SRLL- and FPQQQ and QQIPQQ repetitive motifs. Generally, the ω1,2-gliadins beginning with ARE- are encoded by the D genome, whereas those beginning with ARQ- or KEL- are encoded by the A genome (DuPont et al., 2004). The ω5-gliadins with the SRLL- motif usually are encoded by the B genome (Kasarda et al., 1983; DuPont et al., 2004; Anderson et al., 2009).

Over the years, a number of studies focused on identifying gliadin genes from different wheat species and cultivars (van Herpen et al., 2006; Qi et al., 2009). These studies clearly highlighted the heterogeneity of gene sequences within the α- and γ-gliadin families. However, there are still few complete sequences of ω-gliadin genes. More recently, several studies surveyed the entire complement of gliadin genes within *Aegilops tauschii* and *Triticum urartu*, the diploid progenitors of hexaploid wheat (Zhang et al., 2015; Huo et al., 2017), as well as the reference wheat *T. aestivum* cv. Chinese Spring (Kawaura et al., 2012; Anderson et al., 2013; Noma et al., 2016; Huo et al., 2018a,b). Only a few studies to date have characterized the complement of gliadin genes expressed in wheat cultivars of commercial quality (Altenbach et al., 2010a,b; Li et al., 2014; Wang et al., 2017). Additionally, comprehensive proteomic studies of gliadins have been carried out in only the U.S. wheat Butte 86 (Dupont et al., 2011), the Chinese wheat Xiaoyan 81 (Wang et al., 2017), and Chinese Spring and its aneuploid lines (Kawaura et al., 2018). Assessing the full complement and accumulation levels of gluten proteins in flour from wheat cultivated throughout the world is an important step in understanding how allelic variations in gluten protein genes influence both the functional properties and the immunogenic potential of the flour. Such studies are critical for devising breeding or biotechnology strategies to optimize the quality and healthfulness of wheat flour. In this study, we used 2-dimensional gel electrophoresis (2-DE) combined with tandem mass spectrometry (MS/MS) to determine the gliadin composition of the hexaploid wheat Keumkang, a leading Korean cultivar that is used for both bread and noodle production. The relative amounts of individual proteins accumulated in the flour and the distribution of epitopes for CD and WDEIA in the proteins were also assessed. The work complements an earlier study that characterized the LMW-GS composition of the same cultivar (Lee et al., 2016) and provides the foundation for future studies aimed at improving the functionality and immunogenic potential of Keumkang flour. Additionally, the work contributes to worldwide efforts to compare the gluten protein compositions of wheat cultivars with different end-uses from diverse geographical locations.

## Materials and Methods

### Extraction of Gliadin Protein

Alcohol soluble gliadins were extracted from flour prepared from *T. aestivum* L. cv. Keumkang. Briefly, the flour (100 mg) was shaken in 150 mM NaCl solution (1 mL) for 2 h at room temperature followed by centrifugation at 15,000 *g* and 20°C for 10 min. The supernatant containing albumins and globulins was discarded and the pellets were dissolved in 70% ethanol (1 mL), shaken overnight at room temperature and then centrifuged at 15,000 *g* and 20°C for 10 min. Finally, the supernatant (500 μL) was frozen in liquid nitrogen and stored at -80^o^C. Three separate extractions were performed and analyzed by 2-DE (Supplementary Figure 5).

### Protein Separation Using Two-Dimensional Gel Electrophoresis

To separate individual gliadins using 2-DE, the extracted gliadin fractions were lyophilized, and the resulting pellets were completely dissolved in 150 μL of rehydration buffer containing 7 M urea, 2 M thiourea, 2% (w/v) 3-[(3-cholamidopropyl)-dimethylammonio]-1-propanesulfonate (CHAPS), 0.5% immobilized pH gradient (IPG) buffer (v/v) and 20 mM dithiothreitol (DTT). The amount of protein was determined using the 2D Quant Kit according to the manufacturer’s procedure (GE Healthcare Life Sciences, United States). Gliadin extract (50 mg) was dissolved in 350 μL of rehydration buffer, loaded onto 18 cm IPG strips (GE Healthcare Life Sciences, United States) and then rehydrated in-gel for 15 h at 20^o^C using the IPGphore system (Amersham Biosciences, GE Healthcare Life Sciences, United States). Herein, we used narrow pH range IPG strips of pI 6–11 to yield high resolution of basic α- and γ-gliadins and IPG strips of pI 3–11 NL to resolve acidic omega-gliadins. IEF was carried out for a total of 80 kVh. The IPG gel strip was equilibrated with 75 mM Tris–HCl (pH 8.8) buffer containing 6 M urea, 29.3% glycerol (v/v), 2% SDS (w/v), and 1% DTT (w/v) for 15 min, and then incubated with the same buffer containing 2.5% iodoacetamide instead of DTT for 15 min. Proteins were separated in the second dimension SDS-PAGE gels (12.5%), which were stained with Coomassie Brilliant Blue R-250 for 3 h followed by destaining with 10% methanol (v/v) and 10% acetic acid (v/v) for 3 h. The 2-D gels of each gliadin extract were scanned. Image Master Platinum 7.0 (GE Healthcare Life Sciences, United States) was used to match individual spots across the triplicate gels, determine normalized spot volumes and provide statistical analysis. The average normalized volume of each spot and the standard deviation is shown in Supplementary Table 1.

### Protein Identification by Tandem Mass Spectrometry

For protein identification by MS/MS, individual protein spots were excised from Coomassie-stained 2-D gels, transferred to 96-well plates and subjected to in-gel reduction, alkylation and digestion with thermolysin using a DigestPro (Intavis, Koeln, Germany). Plates containing the peptides were placed in the autosampler of a nanoflow HPLC that was interfaced to an Orbitrap Elite mass spectrometer (Thermo Fisher Scientific, San Jose, CA, United States). Specific details of data acquisition by the instrument were as described previously (Vensel et al., 2014). MS/MS spectra were searched against a database containing *Triticeae* protein sequences downloaded from NCBI on November 1, 2016^1^, cultivar-specific sequences from cv. Butte 86 (Dupont et al., 2011) and a database of common contaminants downloaded on January 30, 2015. Sequences from cv. Butte 86 were included because both cvs. Butte 86 and Keumkang have the *Glu-B3h* and *Glu-D3a* alleles and thus are likely to contain many of the same γ- and ω-gliadins. Two search engines (Mascot and X!Tandem) were used for the analysis and the results were compiled in Scaffold Version 4.7.5. The protein threshold was 99.9%, the minimum number of peptides was four and the peptide threshold was 10 ppm at 95%. MS/MS identifications are shown in Supplementary Table 2. For identification of ω-gliadins, proteins were resolved by 2-DE using an IPG strip with a 3–11 pH range. Because the repetitive sequences of ω-gliadins make individual proteins difficult to distinguish by MS/MS, these spots were digested individually with thermolysin, chymotrypsin, or trypsin and spectral data from the three digests were combined in Scaffold.

### Phylogenic Analyses

To determine the genome assignments of gliadins identified in Keumkang, multiple sequence alignment was performed by Clustal Omega^2^. For α-gliadin analysis, we used α-gliadin sequences from the bread wheat cultivar Xiaoyan 81 including Gli-α1-α8 representing the A genome, Gli-α9-α18 representing the B genome and Gli-α19-α25 representing the D genome (Wang et al., 2017), and α-gliadin sequences from the bread wheat Chinese Spring including nine from the A genome (AB982242, AB982245, AB982249, AB982255, AB982272, AB982277, AB982278, AB982281, AB982288), eight from the B genome (AB982234, AB982236, AB982237, AB982241, AB982267, AB982273, AB982285, AB982286), and nine from the D genome (AB982248, AB982253, AB982256, AB982260, AB982265, AB982276, AB982279, AB982284, AB982293) (Dubois et al., 2016; Noma et al., 2016). For γ-gliadins, we used sequences from the bread wheat cultivar Xiaoyan 81, including three mapped to the A genome (Gli-γ1-γ3), four mapped to the B genome (Gli-γ4 -γ7) and four mapped to the D genome Gli-γ8 -γ11) (Wang et al., 2017). In addition, we used sequences of the first repetitive domain containing CD epitopes from γ-gliadins assigned to the *Gli-A1, Gli-B1*, and *Gli-D1* loci by Salentijn et al. (2012). These sequences were deduced from contigs assembled from 717 transcripts from *T. aestivum* and are listed in Supplementary Table 3. For analysis of ω-gliadins, we used ω-gliadin sequences from the bread wheat cultivar Xiaoyan 81, including one from the A genome (Gli-ω1), two from the B genome (Gli-ω2 and ω3) and two from the D genome (Gli-ω4 and ω5) (Wang et al., 2017). Phylogenic trees of protein families were established with Dendroscope 3 software (ver. 3.5.7).

## Results

**FIGURE 1:**
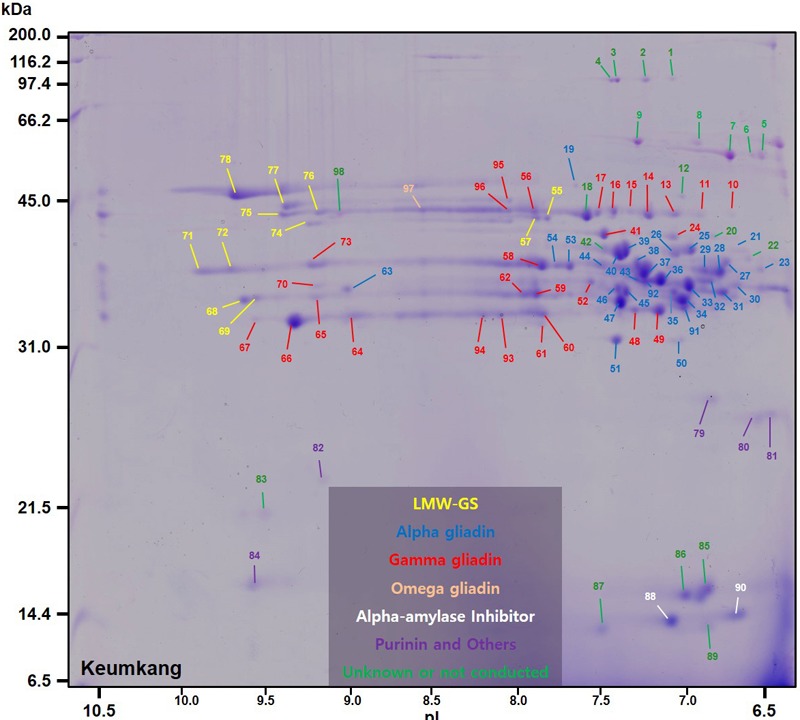
2-dimensional gel electrophoresis (2-DE) separation of proteins in an ethanol-soluble gliadin fraction of cv. Keumkang wheat flour. Spots in which the predominant proteins identified by MS/MS were α-gliadins are labeled in blue, γ-gliadins in red, ω-gliadins in orange, LMW-GS in yellow, α-amylase inhibitors in white, and other non-gluten proteins in purple. Proteins that were not identified are indicated in green.

A total of 98 protein spots were separated by 2-DE from a gliadin extract prepared from wheat flour of cv. Keumkang using a pI range of 6–11 for the first dimension (**Figure 1** and **Table 1**). A number of spots, including spots 45 and 46, spots 39 and 40, spots 37, 38, 43 and 92, spots 34, 35 and 91, spots 25 and 26, and spots 48 and 49 could be resolved in 2-DE using the pI 6–11 IPG strip (**Figure 1**) but not in 2-DE using a wider pI range. Of these, 78 spots were identified by MS/MS. Alpha-gliadins were the predominant proteins in 31 spots (blue), γ-gliadins in 28 spots (red), ω-gliadin in one spot (orange), LMW-GS in 11 spots (yellow), and non-gluten proteins such as purinin, avenin-like protein, α-amylase inhibitor and puroindoline in 7 spots (purple and white).

**Table 1:** Summary of predominant protein types identified by MS/MS in 2-dimensional gel electrophoresis (2-DE) spots of proteins from a gliadin fraction of *Triticum aestivum* cv. Keumkang flour.

Protein type	Spot number	Total spot volume^a^
α-gliadin	31	41.99
γ-gliadin	28	24.84
ω-gliadin	1	0.45
LMW-GS	11	11.95
Non-gluten	7	6.28
Unknown or not conducted	20	14.49
Total	98	100.00

*^*a*^Sum of the volumes of all spots containing a particular protein type as the predominant protein*.

### Alpha-Gliadins

**FIGURE 2:**
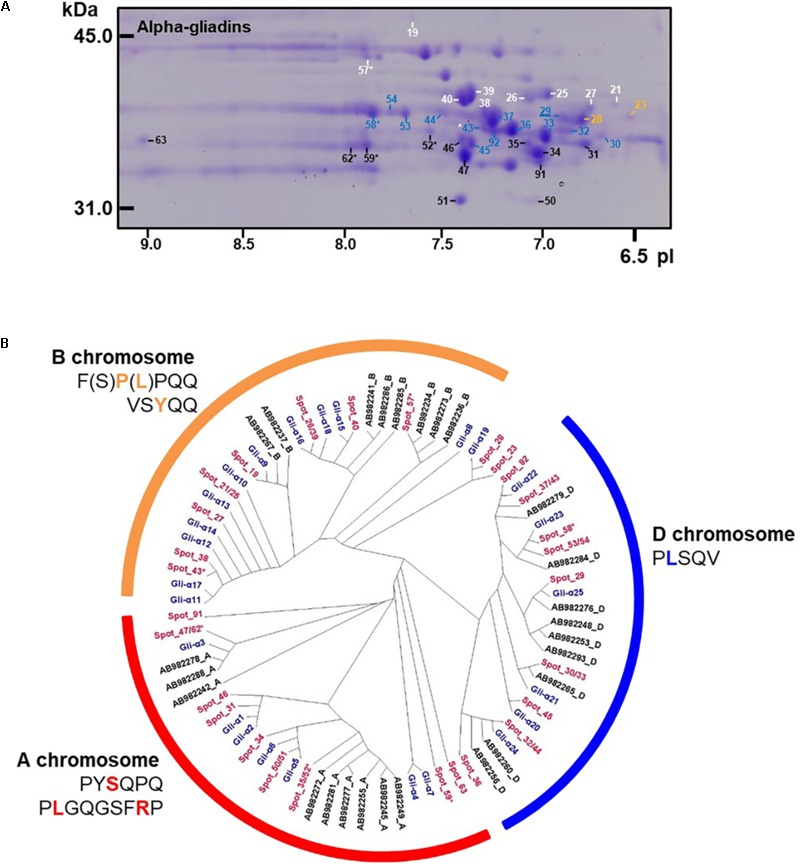
Analysis of α-gliadins from cv. Keumkang flour. **(A)** Region of 2-D gel containing α-gliadins. Proteins encoded by the A genome are labeled in black, B genome in white, and D genome in blue. Spots not assigned to a genome are shown in yellow. Spots in which α-gliadins were identified as minor components are indicated with ^∗^. The underlined spot number denotes the α-gliadin containing seven cysteines. **(B)** Phylogenetic analysis of α-gliadins identified in 2-DE spots from cv. Keumkang (red) with α-gliadins from cvs. Chinese Spring (black) and Xioayan 81 (blue). Sequence motifs identified by Dubois et al. (2016) for each genome are shown.

Alpha-gliadins accounted for more than 40% of the total spot volume of the gliadin fraction (**Table 1**). In addition to being the predominant proteins in 31 spots, α-gliadins were present as minor components in six spots (43, 52, 57, 58, 59, and 62). Most α-gliadin spots clustered between molecular weights of 31 and 45 kDa and pIs between 6.5 and 8.0 (blue labels in **Figure 1**). Spots 33, 34, 36, 37, 39, 40, and 47 were the most abundant α-gliadins in Keumkang (**Figure 2A** and Supplementary Table 1). MS/MS yielded 27 distinct protein sequences for the 37 spots with an average sequence coverage of 78% (**Table 2** and Supplementary Table 2). In a number of cases, spots with similar MWs but different pIs were identified as the same protein sequence. This may be due to charge trains that result from 2-DE or because minor differences in protein sequences are not represented in the database.

**Table 2:** Identification of α-gliadins by 2-DE-MS/MS and protein characteristics.

Locus	Spot number	Predominant protein accession number	Number of unique peptides	Coverage (%)	Number of epitopes for CD^a^					Number of Cys
					DQ2.5-glia-α1a	DQ2.5-glia-α1b	DQ2.5-glia-α2	DQ2.5-glia-α3	DQ8/DQ8.5-glia-α1	
*Gli-A2*		
	31	AKC91211	46	78	1			1		6
	34	AKC91203	61	84	1			1		6
	35	AKB95609	65	89	1			1		6
	52^∗^	AKB95609	24	56	1			1		6
	46	AKC91236	62	84	1			1		6
	47	AKC91207	79	92	1			1		6
	62^∗^	AKC91207	28	76	1			1		6
	50	ABQ52124	31	69	1			1		6
	51	ABQ52124	39	66	1			1		6
	59^∗^	AFX69641	38	70	1			1		6
	63	AKC91229	51	90						6
	91	AFF27490	34	72	1			1		6
*Gli-B2*		
	19	BAM08461	40	63					1	6
	21	BAM08459	30	65					1	6
	25	BAM08459	47	72						6
	26	BU-α23^b^	59	78						6
	39	BU-α23^b^	86	86						6
	27	BAM08455	62	85						6
	38	BU-α12^b^	53	74						6
	40	BU-α8^b^	85	88						6
	43^∗^	BAM08454	31	87						6
	57^∗^	BAS02443	20	57					1	6
*Gli-D2*		
	29	ACX71610	51	85	1	1	1		1	7
	30	BU-α4^b^	80	87	1		1	1	1	6
	33	BU-α4^b^	95	87	1		1	1	1	6
	32	AFX69619	57	70	1		1	1		6
	44	AFX69619	37	74	1		1	1		6
	36	BU-α10^b^	87	89	1		1	1		6
	37	BU-α3^b,c^	87	89	1	1	3	1	1	6
	43	BU-α3^b,c^	49	76	1	1	3	1	1	6
	45	AFQ13463	77	91	1	1	1	1	1	6
	53	BU-α1^b,c^	62	91	1	1	3	1	1	6
	54	BU-α1^b,c^	59	92	1	1	3	1	1	6
	58^∗^	ABS72161^c^	36	91	1	1	3	1	1	6
	92	ABD85199^c,d^	38	65	1	1	3	1	1	6
Unknown		
	23	AFX69625	10	27	1	1	1	1		6
	28	AFX69606	55	73	1			1		6

*^∗^Indicates spots containing α-gliadins as minor components. ^*a*^Core sequences of CD-relevant epitopes include DQ2.5-glia-α1a (PFPQP****Q****LPY), DQ2.5-glia-α1b (PYPQP****Q****LPY), DQ2.5-glia-α2 (PQP****Q****LPYPQ), DQ2.5-glia-α3 (FRP****Q****QPYPQ), and DQ8-glia-α1/DQ8.5-glia-α1 (****Q****GSFQPSQ****Q****). The bold residues represent target sequences for tTG deamidation that are important for recognition by T cells (Sollid et al., 2012). ^*b*^Sequences were obtained by assembling ESTs from T. aestivum cv. Butte 86 and are reported in Dupont et al. (2011). ^*c*^Also contains 33-mer toxic peptide. ^*d*^Identical to BU-α3*.

A phylogenetic analysis was performed with the amino acid sequences of α-gliadins from Chinese Spring and Xiaoyan 81 to determine the chromosomal origins of α-gliadins in Keumkang, (**Figure 2B**). The results showed that the α-gliadins in Keumkang spots 31, 34, 35/52, 46, 47/62, 50/51, 59, 63, and 91 clustered with Chinese Spring and Xiaoyan 81 sequences mapped to the A genome, those in spots 19, 21/25, 26/39, 27, 38, 40, 43, and 57 clustered with sequences mapped to the B genome and those in spots 29, 30/33, 32/44, 36, 37/43, 45, 53/54, 58, and 92 clustered with sequences mapped to the D genome (**Figure 2B** and **Table 2**). The chromosomal assignments of two spots (23 and 28) were unclear, although both were most similar to α-gliadins assigned to the D genome. Overall, assignments were consistent with the presence of genome-specific motifs described by Dubois et al. (2016) (Supplementary Figure 1). All sequences assigned to the B genome also contained the CSTT motif identified by Wang et al. (2017). It is also interesting that α-gliadins encoded by the A, B, and D genomes were located at the lower, center, and upper parts, respectively, of the α-gliadin region of the 2-D gel (**Figure 2A**).

The largest number of CD epitopes were found in α-gliadins encoded by the D genome (**Table 2** and Supplementary Figure 1). All had one DQ2.5-glia-α1a and one to three DQ2.5-glia-α2 epitopes. All but one of the D-encoded α-gliadins also contained the DQ2.5-glia-α3 epitope, five contained the DQ2.5-glia-α1b epitope and seven contained the DQ8-glia-α1 epitope. Three α-gliadins, BU-α1, BU-α3, and ABS72161, also contained the 33-mer peptide LQLQPFPQPQLPYPQPQLPYPQPQLPYPQPQPF with multiple overlapping CD epitopes that has been shown to be resistant to proteolytic cleavage and particularly toxic to CD patients (Shan et al., 2002). BU-α1 and BU-α3 were the predominant proteins in five spots (37, 43, 53, 54, and 92) that accounted for 15.9% of the α-gliadins and 6.7% of the total gliadin fraction (Supplementary Table 4). Alpha-gliadins encoded by the B genome contained the fewest CD epitopes. Three of the eight α-gliadins from the B genome each had a single DQ8-glia-α1 epitope while the rest had none of the identified sequences. All but one of the α-gliadins encoded by the A genome contained single copies of both the DQ2.5-glia-α1 and the DQ2.5-glia-α3 epitopes. Proteins encoded by the D genome encompassed 16.6% of the total spot volume of the gliadin fraction, while those encoded by the A and B genomes encompassed 12.0 and 11.4% of the spot volume, respectively (Supplementary Table 4).

Only one of the α-gliadins identified in Keumkang contained seven cysteines instead of the usual six (**Table 2**). This protein was identified in a relatively minor spot (29). This is not surprising since the extra cysteine would be expected to enable the protein to link into the glutenin polymer. Thus, it would be expected that the bulk of this protein would be found in a glutenin fraction rather than a gliadin fraction.

### Gamma-Gliadins

**FIGURE 3:**
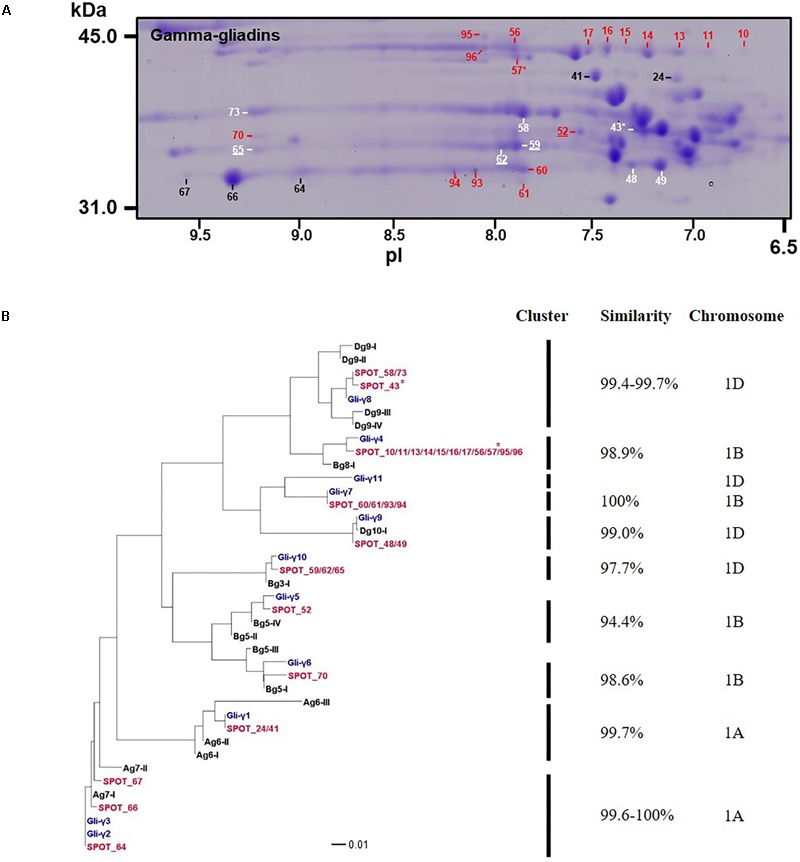
Analysis of γ-gliadins from cv. Keumkang flour. **(A)** Region of 2-D gel containing γ-gliadins. Proteins encoded by the A genome are labeled in black, B genome in red, and D genome in white. Spots in which γ-gliadins were identified as minor components are indicated with ^∗^. Spot numbers that are underlined denote γ-gliadins containing nine cysteines. **(B)** Phylogenetic analysis of γ-gliadins identified in 2-DE spots from cv. Keumkang (red) with γ-gliadins from Xiaoyan 81 (blue) and γ-gliadin sequences deduced from contigs assembled from 717 *Triticum aestivum* γ-gliadin transcripts (black, Supplementary Table 3). The similarity of sequences within each cluster is shown along with the chromosomal locations determined for γ-gliadins from cv. Xioayan 81.

Gamma-gliadins accounted for nearly 25% of the total gliadin fraction (**Table 1**). Twelve γ-gliadins were identified as the predominant proteins in 28 spots or as minor proteins in two spots (43 and 57) (red labels in **Figure 1**, **Table 3**, and Supplementary Table 2). The γ-gliadin spots were scattered between molecular weights of 31 and 45 kDa and pIs of 6.5 and 9.5 (**Figure 3A**). Seven of the γ-gliadins identified in cv. Keumkang matched sequences from cv. Butte 86, a cultivar that shares some of the same alleles. Two of the other sequences were either identical to partial γ-gliadin sequences from Butte 86 (AAD30440) or were simply missing the sequences for the signal peptide (ACI04085) (**Table 3**). Average MS/MS coverage for the γ-gliadins was 56.1% although some were as high as 83% (**Table 3**). Spot 66, identified as BU-γ6, was the most abundant γ-gliadin spot in the fraction, accounting for 4.6% of the total spot volume (**Figure 1, Table 3** and Supplementary Table 4). BU-γ5 was identified in eleven spots (10, 11, 13, 14, 15, 16, 17, 56, 57, 95, and 96) with similar molecular weights (∼44 kDa) but different pIs that most likely result from charge trains in 2-DE (**Figure 1** and **Table 3**). Together these spots accounted for 4.6% of the total volume (Supplementary Table 4). BU-γ2 was identified in two spots that accounted for 3.3% of the total proteins and BU-γ4 was detected as the predominant protein in three spots (59, 62, and 65) that accounted for 3.0% of the spot volume (**Table 3** and Supplementary Table 4).

**Table 3:** Identification of γ-gliadins by 2-DE-MS/MS and protein characteristics.

Locus	Spot number	Predominant protein accession Number	Number of unique peptide	Coverage (%)	Number of epitopes for CD^a^								Number of Cys
					γ1	γ2	γ3	γ4a	γ4b	γ4c	γ4d	γ5	
*Gli-A1*													
	24	AGJ50340	19	39		1				6		2	8
	41	AGJ50340	38	66		1				6		2	8
	64	ACJ03455	31	75	1					4			8
	66	BU-γ6^b^	54	61	1					4			8
	67	ACJ03500	16	46	1					4			8
*Gli-B1*													
	10	BU-γ5^b^	19	46	1					8		4	8
	11	BU-γ5^b^	13	26	1					8		4	8
	13	BU-γ5^b^	32	64	1					8		4	8
	14	BU-γ5^b^	44	54	1					8		4	8
	15	BU-γ5^b^	24	46	1					8		4	8
	16	BU-γ5^b^	55	56	1					8		4	8
	17	BU-γ5^b^	24	51	1					8		4	8
	56	BU-γ5^b^	47	70	1					8		4	8
	57^∗^	BU-γ5^b^	22	44	1					8		4	8
	95	BU-γ5^b^	13	26	1					8		4	8
	96	BU-γ5^b^	13	27	1					8		4	8
	60	BU-γ7^b^	48	75	1	1	1			2			8
	61	BU-γ7^b^	30	61	1	1	1			2			8
	93	BU-γ7^b^	27	48	1	1	1			2			8
	94	BU-γ7^b^	17	49	1	1	1			2			8
	70	BU-γ9^b^	41	49	1	1			1	4			8
	52	BU-γ10^b^	35	52	1	1			1	4	1		9
*Gli-D1*													
	43^∗^	ACI04085^c^	21	62	1	1				6		3	8
	58	BU-γ2^b^	54	83	1	1				6		3	8
	73	BU-γ2^b^	57	80	1	1				6		3	8
	48	AAD30440^d^	38	72	1	1	1	1		1		1	8
	49	AAD30440^d^	58	76	1	1	1	1		1		1	8
	59	BU-γ4^b^	39	56	1	1				3	1		9
	62	BU-γ4^b^	39	67	1	1				3	1		9
	65	BU-γ4^b^	39	56	1	1				3	1		9

*^∗^Indicates spots containing γ-gliadins as minor components. ^*a*^Core sequences of CD-relevant epitopes include γ1, DQ2.5-glia-γ1/DQ8-glia-γ1 (PQQSFP****Q****QQ); γ2, DQ2.5-glia-γ2 (IQP****Q****QPAQL); γ3, DQ2.5-glia-γ3/DQ8-glia-γ1b (QQP****Q****QPYPQ); γ4a, DQ2.5-glia-γ4a (SQP****Q****Q****Q****FPQ); γ4b, DQ2.5-glia-γ4b (PQP****Q****Q****Q****FPQ); γ4c, DQ2.5-glia-γ4c (QQP****Q****QPFPQ)/DQ8-glia-γ1a (****Q****QPQQPFPQ); γ4d, DQ2.5-glia-γ4d (PQP****Q****QPFCQ); γ5, DQ2.5-glia-γ5 (QQPFP****Q****QPQ). The bold residues represent target sequences for tTG deamidation that are important for recognition by T cells (Sollid et al., 2012). ^*b*^Sequences were obtained by assembling ESTs from T. aestivum cv. Butte 86 and are reported in Dupont et al. (2011). ^*c*^Identical to BU-γ2. ^*d*^Identical to partial sequence BU-γ11*.

Phylogenetic analysis (**Figure 3B** and **Table 3**) showed that proteins contained in five spots (24, 41, 64, 66, and 67) are likely encoded by the A genome. Spots 24 and 41 had high similarities with Gli-γ1 (99.7%) while spots 64, 66, and 67 were very similar to Gli-γ2 (99.6–100%). Gliadins identified in eight spots (43, 48, 49, 58, 59, 62, 65, and 73) clustered with sequences characteristic of the D genome. Proteins in spots 43, 58, and 73 were very similar to Gli-γ8 (99.4–99.7%), proteins in spots 48 and 49 were similar to Gli-γ9 (99.0%) and proteins in spots 59, 62, and 65 were similar to Gli-γ10 (97.7%), all encoded by the *Gli-D1* loci (**Figure 3B**). In addition, BU-γ5, identified in 11 spots (10, 11, 13, 14, 15, 16, 17, 56, 57, 95, and 96), BU-γ7 identified in four spots (60, 61, 93, and 94), and BU-γ9 and BU-γ10, identified in spots 70 and 52, respectively, clustered with B genome-specific epitope sequences and were similar to Gli-γ4 (98.9%), Gli-γ7 (100%), Gli-γ6 (98.6%), and Gli-γ5 (94.4%), respectively, all encoded at the *Gli-B1* loci. Overall, γ-gliadins encoded by the A and B genomes comprised 7.6 and 7.7% of the total spot volume, while those encoded by D genome comprised 9.5% (Supplementary Table 4).

Four spots identified as either BU-γ4 (59, 62, 65) or BU-γ10 (52) contained γ-gliadins with nine cysteines instead of the usual eight (**Table 3** and Supplementary Figure 2). Of these, spot 59 was a moderately abundant spot while the others were relatively minor. Spots 59, 62 and 65 were encoded by D genome while Spot 52 was encoded by the B genome.

### Omega-Gliadins

**FIGURE 4:**
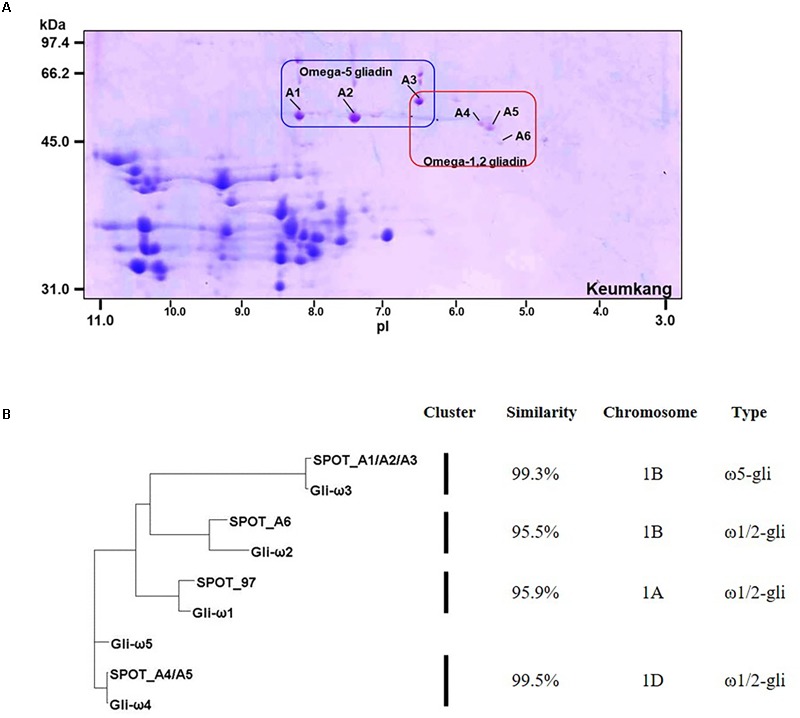
Analysis of ω-gliadins from cv. Keumkang flour. **(A)** Region of 2-D gel containing ω-gliadins. 2-DE was conducted with a pI range from 3–11. Proteins in spots A1, A2, and A3 were identified by MS/MS as ω5-gliadins and proteins in spots A4, A5, and A6 were identified by MS/MS as ω1,2-gliadins. **(B)** Phylogenetic analysis of ω-gliadins identified in 2-DE spots from cv. Keumkang with ω-gliadins from cv. Xiaoyan 81. The similarity of sequences within each cluster is shown along with the chromosomal locations determined for ω-gliadins from Xioayan 81. The type of ω-gliadin is also indicated.

Omega-gliadins generally are more acidic than many of the α- and γ-gliadins and are found between 46 and 66 kDa in 2-DE (Supplementary Figure 6). Although there were several spots (5, 6, 7, 8, and 9) in this region of the gel, none of these yielded MS/MS identifications (**Figure 1** and Supplementary Table 2). Only one ω-gliadin was identified and this was in a minor spot (97) with a molecular weight of about 40 kDa and pI of about 8.5 (**Figure 1** and **Table 4**). The spot was identified as ω1,2-gliadin ADF58069 beginning with the N-terminal sequence ARQL- and containing 12 copies of a PQQPFP motif. Omega-gliadins are particularly difficult to identify by MS/MS. Because of their highly repetitive sequences, many of the genes are difficult to clone. Thus, there is a lack of good sequences for ω-gliadins in databases used to analyze spectral data. To identify the ω-gliadins from Keumkang, an additional experiment was conducted in which the gliadins were separated by 2-DE using a wider pI range of 3–11. Six spots (A1–A6) were excised from triplicate gels, digested separately with trypsin, chymotrypsin, or thermolysin and analyzed by MS/MS (**Figure 4**). Omega-gliadins were identified in all six spots with MS/MS coverages that ranged from 24 to 71% (**Table 4**). Spots A1 and A2 were identified as ω5-gliadin BAE20328 beginning with the N-terminal sequence SRLL- and containing 28 copies of a FPQQQ motif and 10 copies of a QQIPQQ motif. Spot A3 was identified as the closely-related ω5-gliadin CAR82267 beginning with GRLL- and containing 23 copies of a FPQQQ motif and nine copies of a QQIPQQ motif (Supplementary Figure 3). Spots A4 and A5 were identified as ω1,2-gliadin BAN29067 beginning with AREL- and containing 18 copies of a PQQPFP motif. Spot A6 was identified as CAI78903 with an N-terminal sequence of SRLL- that is typical of ω5-gliadins. However, the protein contains 12 copies of the ω1,2-gliadin motif PQQPFP. Phylogenetic analysis of these omega gliadins with five ω-gliadins from cv. Xiaoyan 81 that were mapped using deletion lines suggest that spot 97 is encoded by the A genome, spots A1, A2, A3, and A6 are encoded by the B genome and spots A4 and A5 are encoded by the D-genome (**Figure 4B** and **Table 4**).

**Table 4:** Identification of ω-gliadins by 2-DE-MS/MS and protein characteristics.

Protein type/locus	Spot number	Accession no.	Number of unique peptide	Coverage (%)	N-terminal sequence	CD epitopes^1^				WDEIA epitopes^2^			
						ω1	ω2	γ4c	γ5	WD-1	WD-2	WD-3	WD-4
ω-1,2/*Gli-A1*													
	97	ADF58069	17	42	ARQL-			1	3				
ω-5/*Gli-B1*													
	A1	BAE20328	42	64	SRLL-					4	12	1	
	A2	BAE20328	33	58	SRLL-					4	12	1	
	A3^∗^	CAR82267^3^	64	71	GRLL-					4	17	1	1
ω-1,2/*Gli-B1*													
	A6	CAI78903	15	37	SRLL-			2	2				
ω-1,2/*Gli-D1*													
	A4	BAN29067	10	24	AREL-	1	1	7	9				
	A5	BAN29067	15	48	AREL-	1	1	7	9				

*^∗^Indicates spots containing α-gliadins as minor components. ^*1*^CD-relevant epitopes include ω1, DQ2.5-glia-ω1 (PFPQP****Q****QPF); ω2, DQ2.5-glia-ω2 (PQP****Q****QPFPW); γ4c, DQ2.5-glia-γ4c (QQP****Q****QPFPQ)/DQ8-glia-γ1a (****Q****QPQQPFPQ); γ5, DQ2.5-glia-γ5 (QQPFP****Q****QPQ). The bold residues represent target sequences for tTG deamidation that are important for recognition by T cells (Sollid et al., 2012). ^*2*^WDEIA epitopes include QQIPQQQ (WD-1), QQFPQQQ (WD-2), QQSPQQQ (WD-3) and QQSPEQQ (WD-4) (Matsuo et al., 2004). ^*3*^contains a single cysteine.*

The ω5-gliadins contained multiple epitopes shown to be dominant in WDEIA, particularly QQIPQQQ and QQFPQQQ (**Table 4** and Supplementary Figure 4). Of the ω1,2-gliadins, only BAN29067 contained the CD epitopes DQ2.5-glia-ω1 and DQ2.5-glia-ω2 described by Sollid et al. (2012). However, all ω1,2-gliadins contained several CD epitopes characteristic of γ-gliadins. In particular, BAN29067 contained seven copies of DQ2.5-glia-γ4c/DQ8-glia-γ1a and nine copies of DQ2.5-glia-γ5 (Supplementary Figure 3).

Of the ω-gliadins identified in Keumkang, only CAR82267 in spot A3 contained a single cysteine residue close to the C-terminus that would enable the protein to link into the glutenin polymer (**Table 4**).

### LMW-GS

Eleven 2-DE spots comprising 12% of the total spot volume of the gliadin fraction were identified as LMW-GS (**Figure 1** and **Table 5**). With the exception of spot 78, all were relatively minor spots in this fraction. Five other spots contained LMW-GS as minor components. An i-type LMW-GS beginning with the N-terminal sequence ISQQQ- was identified as the predominant protein in spot 77 and as a minor component of spot 96. Two different s-type LMW-GSs beginning with the N-terminal sequence SHIP- were identified in spots 75 and 78. Seven different m-type LMW-GS beginning with METSHIP-, METSRV-, METSCIC-, METSCIP-, or METRCIP were identified as the predominant proteins in eight spots (55, 57, 68, 69, 71, 72, 74, and 76) and as minor components in four spots (16, 46, 56, and 95). Previously, we cloned 33 genes from genomic DNA of wheat cv. Keumkang, classified the genes into nine haplotypes and linked them to their protein products in 2-DE. Haplotypes included one *Glu-A3* (*GluA3-13*), two *Glu-B3* (*GluB3-33*, and *-43*), and six *Glu-D3* (*GluD3-11, -21, -31, -42, -5*, and *-6*) (Lee et al., 2016). In this study, proteins corresponding to each haplotype were identified in the gliadin fraction from Keumkang (**Table 5**).

**Table 5:** Identification of LMW-GS by 2-DE-MS/MS and protein characteristics.

Predominant protein	Spot number	Accession no.	Number of unique peptide	Coverage (%)	N-terminal sequence	Similar protein from cv. Keumkang	% Identity	Gene /Allele^a^
*LMW-GS Glu-A3*		
	77	AAS10189	62	87	ISQQQ-	ALN96379	99.5	*GluA3-13*
	96^∗^	AAS10187	13	40	ISQQQ-	ALN96379	99.2	*GluA3-13*
*LMW-GS Glu-B3*		
	74	AEI00658	53	78	METSHIP-	ALN96383	99.4	*GluB3-43*
	78	ACA63869	86	84	SHIP-	ALN96381	99.7	*GluB3-33*
*LMW-GS Glu-D3*		
	16^∗^	BU-LMW-GS PCR#2^b^	31	56	METSRV-	ALN96388	100	*GluD3-11*
	55	BU-LMW-GS PCR#2^b^	49	74				
	57	BU-LMW-GS PCR#2^b^	68	81				
	46^∗^	AAP44989	29	70	METSCIS-	ALN96400	100	*GluD3-42*
	56^∗^	ABY58134	36	70	METSHIP-	ALN96403	99.2	*GluD3-5*
	76	ABY58134	63	78				
	68	AAV92011	53	82	METSCIP-	ALN96404	99.3	*GluD3-6*
	69	AAV92011	52	73				
	71	BU-LMW-GS PCR#3^b^	63	80	METRCIP-	ALN96395	98	*GluD3-21*
	72	BU-LMW-GS PCR#3^b^	80	79				
	95^∗^	ACY08820	12	41	METSHIP-	ALN96403	99.4	*GluD3-31*
	75	ABC84366	58	83	SHIP-	ALN96399	100	*GluD3-5*

*^∗^Indicates spots containing LMW-GS as minor components. ^*a*^From Lee et al. (2016). ^*b*^DNA sequence amplified from RNA of T. aestivum cv. Butte 86. Deduced protein sequence can be found in Supplementary Table 2.*

### Non-gluten Proteins

A number of non-gluten proteins also were identified in the gliadin fraction (**Figure 1**). Purinins were detected as predominant proteins in three spots (79, 80, and 81) that accounted for 2.1% of total spot volume (**Table 6** and Supplementary Table 1). The spots were resolved at about 25 kDa and had pIs between 6.5 and 7.0 (purple labels in **Figure 1**). Two spots (89 and 90) with molecular weights around 14.4 kDa and pIs between 6.5 and 7.0 were identified as α-amylase inhibitors (white labels in **Figure 1** and **Table 6**). These spots, accounted for 3.0% of the spot volume (Supplementary Table 1). Several other spots in this area of the gel (85, 86, 87, and 89) did not yield MS/MS identifications but are likely to be other α-amylase inhibitors. An avenin-like protein was identified in spot 82 at about 21.5 kDa between pIs of 9.0 and 9.5 and a puroindoline was found in spot 84 at about 15 kDa and 9.5 pI (**Figure 1** and **Table 6**). Within the gliadin region of the gel, a number of minor spots (12, 20, 22, 42, and 98) as well as one fairly abundant spot (18) did not yield MS/MS identifications in the analysis (green labels in **Figure 1**).

**Table 6:** Identification of non-gluten proteins by 2DGE-MS/MS.

Predominant protein	Spot number	Accession no.	Number of unique peptide	Coverage (%)
Purinin	79	Purinin BU-2 (BQ804660)^a^	7	34
	80	Purinin BU-1 (BQ838917)^a^	7	34
	81	Purinin BU-3 (BQ804472)^a^	16	51
Avenin-like protein	48^∗^	AHA61701	35	57
	82	Avenin-like BU-2^a^	12	43
Alpha-amylase inhibitor	89	CDM81434 (dimeric)	6	38
	90	CAA39099 (tetrameric CM2)	6	38
Puroindoline	84	AGH06195	6	16

*^∗^Indicates spots with non-gluten proteins as minor components. ^*a*^Sequences obtained by assembling ESTs from T. aestivum cv. Butte 86 and are reported in Dupont et al. (2011)*.

## Discussion

Because of the considerable sequence variation in α-, γ-, and ω-gliadins both within wheat cultivars and among different cultivars, detailed knowledge about the composition of gliadin genes and proteins in individual wheat cultivars is critical for understanding how these proteins contribute to both the functional properties and the immunogenic potential of the flour. The current study reports 39 gliadin sequences identified by MS/MS in 66 individual 2-DE spots from a gliadin fraction from the Korean wheat cultivar Keumkang. Proteins identified include 23 α-gliadins (**Table 2**), 11 γ-gliadins (**Table 3**), and 5 ω-gliadins (**Table 4**). This number is in line with the findings of Wang et al. (2017) who reported the sequences of 25 α-, 11 γ-, and 5 ω-gliadins expressed in the Chinese cultivar Xioayan 81 and Dupont et al. (2011) who used MS/MS to identify 23 α-, 13 γ-, and 7 ω-gliadins in the U.S. cultivar Butte 86. Recently, annotation of genome sequence data from the reference wheat Chinese Spring combined with analysis of transcriptomic data yielded similar numbers of expressed gliadin genes as well as 22, 3, and 13 α-, γ-, and ω-gliadin pseudogenes, respectively (Huo et al., 2018a,b). Taken together, data from genome sequencing, transcriptomic and proteomic analyses indicate that the complexity of the gliadin gene families in hexaploid wheat may be considerably less than originally reported (Sabelli and Shewry, 1991; Anderson and Greene, 1997). Nonetheless, the genome-wide characterization of gliadins remains challenging largely because of the sequence similarities of family members.

In this study, the average MS/MS sequence coverage for the α- and γ-gliadins was 68% with coverages for many α-gliadins greater than 85%. This is higher than might have been expected since the complete complement of gliadin protein sequences from Keumkang was not present in the database used for interrogation of spectral data. However, the numbers of gliadin sequences in public databases have increased dramatically in recent years and gliadin sequences from the cultivar Butte 86 were included since Butte 86 and Keumkang share some of the same alleles. It is not surprising then that the best match of γ-gliadins from the B and D genomes were to sequences from Butte 86.

Alpha-gliadins comprised the largest proportion of the gliadin fraction in Keumkang. Of these, α-gliadins encoded by the D genome were the most abundant, encompassing nearly 40% of the total while those encoded by the A and B genome encompassed 29 and 27%, respectively (Supplementary Table 4). This is of significance because α-gliadins encoded by the D genome contain more CD epitopes than those encoded by the other genomes (**Table 2**). In fact, all α-gliadins encoded by the D genome contained copies of the DQ2.5-glia-α1a and DQ2.5-glia-α2 epitopes found to be immunodominant in CD patients by Tye-Din et al. (2010). Additionally, two of the eight α-gliadins encoded by the D genome contained the 33-mer peptide that has been found to be particularly toxic and these were highly expressed, comprising over 40% of the D genome α-gliadins in Keumkang. It is also worth noting that seven of the nine α-gliadins encoded by the B genome in Keumkang and one of the nine α-gliadins encoded by the A genome had none of the identified CD epitopes. These proteins comprised only about 24% of the α-gliadins in Keumkang.

Gamma-gliadins encoded by the D genome also were more abundant in Keumkang with 38% from the D genome and 31% from each of the A and the B genomes (Supplementary Table 4). However, CD epitopes were distributed more evenly among γ-gliadins from the different genomes, so the proportions of proteins from each genome may not be as important as for α-gliadins (**Table 3** and Supplementary Figure 2). Nonetheless, it is interesting two of the γ-gliadins that contain the greatest numbers of CD epitopes, BU-γ5 containing eight DQ2.5-glia-γ4c/ DQ8-glia-γ1a epitopes, four DQ2.5-glia-γ5 epitopes, and one DQ2.5-glia-γ1/ DQ8-glia-γ1 epitope, and BU-γ2, containing six DQ2.5-glia-γ4c/ DQ8-glia-γ1a epitopes, three DQ2.5-glia-γ5 epitopes, one DQ2.5-glia-γ1/ DQ8-glia-γ1 epitope, and one DQ2.5-glia-γ2 epitope, accounted for more than 31% of the total γ-gliadins in Keumkang.

Of the ω-gliadins identified in Keumkang, those encoded by the D genome contained the greatest number of CD epitopes and included both the DQ2.5-glia-ω1 and the immunodominant DQ2.5-glia-ω2 epitopes as well as seven copies of each of the two γ-gliadin epitopes DQ2.5-glia-γ4c/DQ8-glia-γ1a and DQ2.5-glia-γ5 (**Table 4** and Supplementary Figure 2). Omega-gliadins encoded by the A genome contained only a few of the γ-gliadin CD epitopes while those encoded by the B genome did not contain CD epitopes but contained multiple copies of epitopes that trigger the serious food allergy WDEIA.

Similarities among the sequences of the γ- and ω-gliadin proteins identified in Korean cultivar Keumkang and the Chinese cultivar Xiaoyan 81 are striking (**Figures 3B, 4**). The same is true for most of the α-gliadins (data not shown). Since most CD and WDEIA epitopes contain high proportions of glutamine and proline, it is easy to see that even small differences in the sequences of these proteins among cultivars can affect the number of CD epitopes and impact the immunogenic potential of the flour. The proportions of the different proteins in the flour also must be considered (Juhász et al., 2012). However, quantitative proteomic data for Xioayan 81 was not reported so these comparisons cannot be made. It is perhaps more challenging to hypothesize how small changes in protein sequences might impact flour quality. In this respect, the relative proportions of gliadins containing odd numbers of cysteine residues may be important since these proteins are likely to link into the glutenin polymer and affect polymer size by acting as chain terminators. In Keumkang, one α-gliadin, two γ-gliadins and one ω-gliadin contained an odd number of cysteine residues. It was not possible to quantify the amounts of these proteins in this study since the majority of these proteins should be found in the glutenin fraction rather than the gliadin fraction. But clearly it will be important to look for these specific proteins in both the glutenin fraction as well as total protein fractions in future proteomics experiments of flour from Keumkang.

Future efforts to improve wheat will benefit from genome-wide characterization of gliadin genes, transcripts and proteins in a wide range of wheat cultivars. The availability of the draft genome sequence from the reference cultivar Chinese Spring now makes it possible to conduct targeted gene capture experiments for high throughput genome sequencing of regions harboring gliadin genes in wheat cultivars grown throughout the world for different end-uses. Improvements in DNA sequencing technology will hasten transcriptomic studies in the same cultivars and facilitate the accurate identification of individual gliadins by MS/MS and the development of targeted proteomics approaches to detect and quantify the levels of specific immunogenic peptides in wheat flour (van den Broeck et al., 2015; Martinez-Esteso et al., 2016; Bromilow et al., 2017; Schalk et al., 2017). This knowledge will make it possible to design strategies to alter protein composition of the flour through molecular breeding or genome editing and improve the functionality and healthfulness of wheat.

## Author Contributions

KC, H-RB, Y-RJ, WV, and AS-B designed and carried out the experiments. KC analyzed the results and wrote the manuscript. SA and J-YL contributed scientific advice and corrected the manuscript. S-HL and MK contributed scientific advice, critical reading, revision, and editing of the manuscript. All authors read and approved the manuscript.

## Conflict of Interest Statement

The authors declare that the research was conducted in the absence of any commercial or financial relationships that could be construed as a potential conflict of interest.

## Footnotes

1 www.ncbi.nlm.nih.gov

2 http://www.ebi.ac.uk/Tools/msa/clustalo/
