# Supplementary material for: Proteomic Profiling and Epitope Analysis of the Complex α-, γ-, and ω-Gliadin Families in a Commercial Bread Wheat
Source: Front Plant Sci. 2018 Jun 19;9:818. doi: 10.3389/fpls.2018.00818 (PMC6018075; doi:10.3389/fpls.2018.00818)
Supplement: Supplementary file 1 [file Presentation_1.PPTX]

## Slide 1
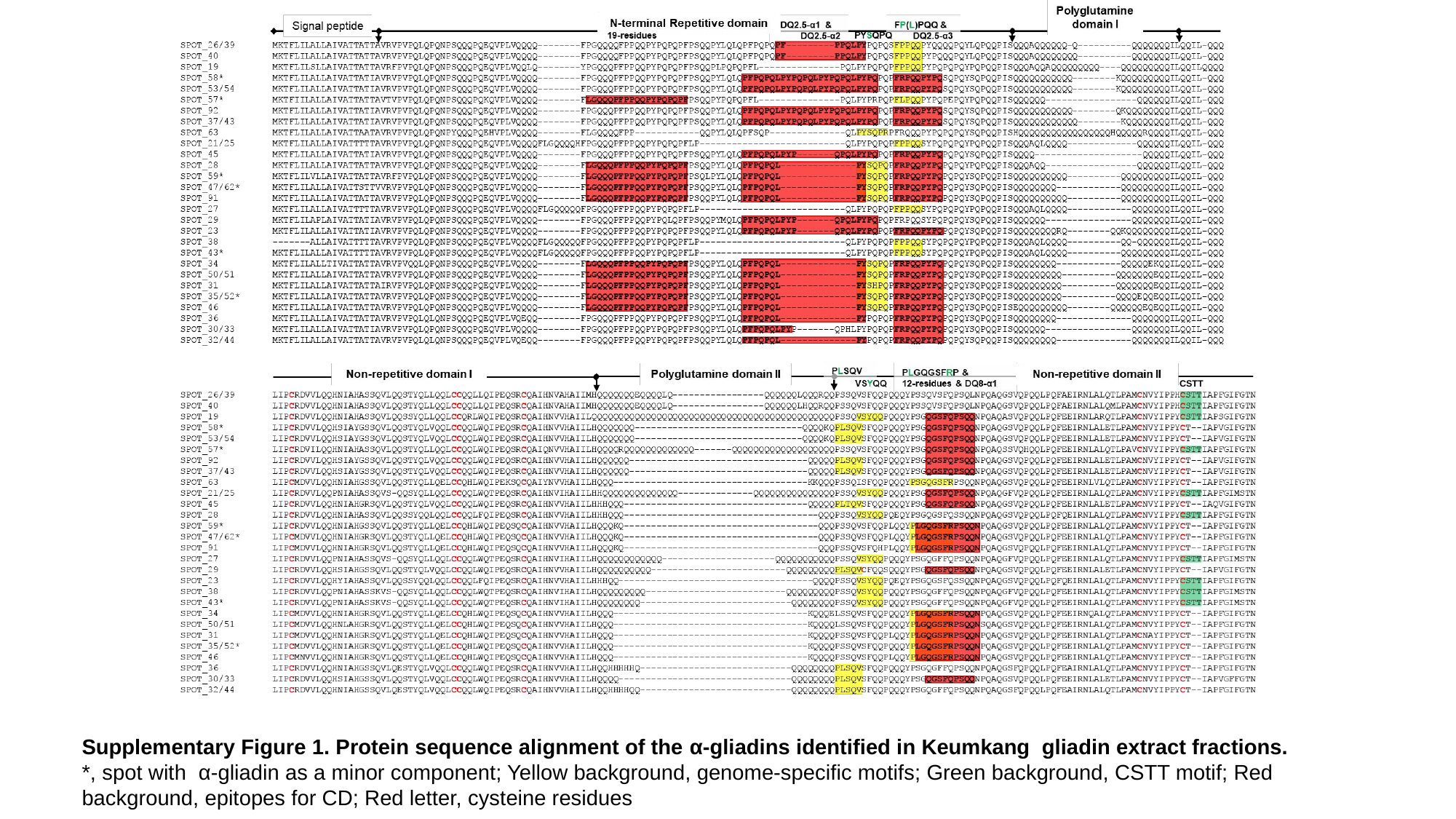

CSTT
Supplementary Figure 1. Protein sequence alignment of the α-gliadins identified in Keumkang gliadin extract fractions.
*, spot with α-gliadin as a minor component; Yellow background, genome-specific motifs; Green background, CSTT motif; Red background, epitopes for CD; Red letter, cysteine residues

## Slide 2
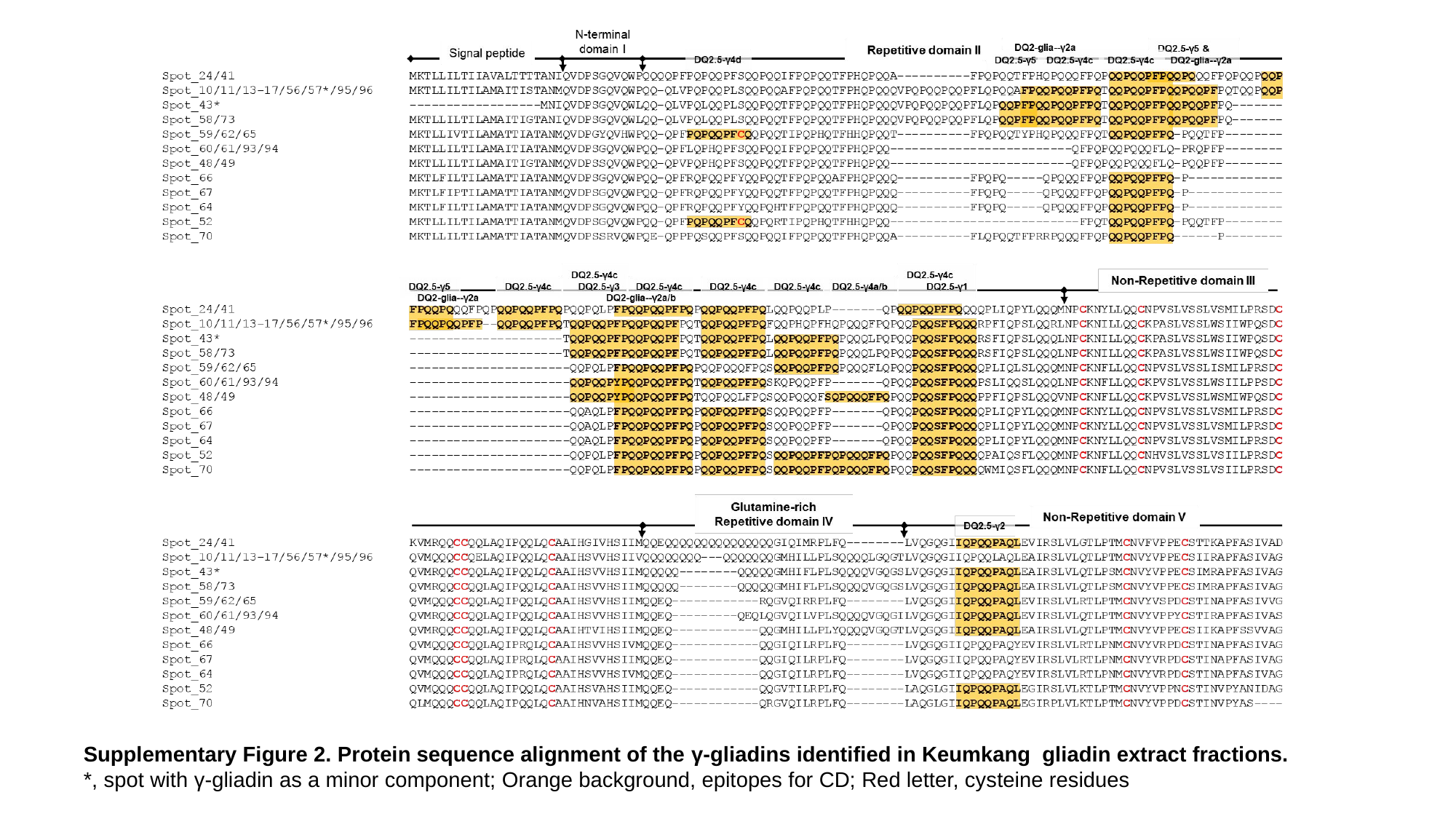

Supplementary Figure 2. Protein sequence alignment of the γ-gliadins identified in Keumkang gliadin extract fractions.
*, spot with γ-gliadin as a minor component; Orange background, epitopes for CD; Red letter, cysteine residues

## Slide 3
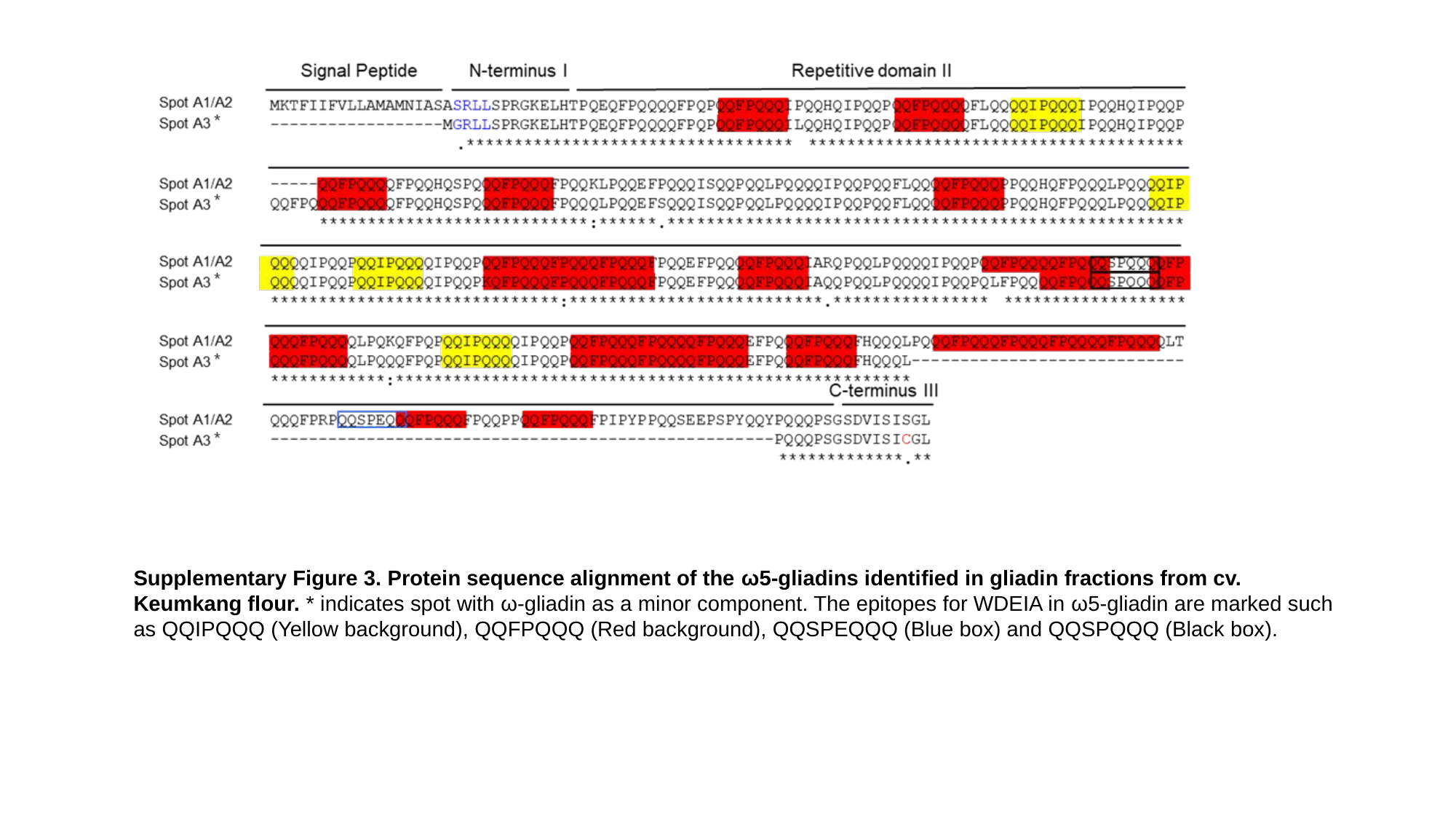

Supplementary Figure 3. Protein sequence alignment of the ω5-gliadins identified in gliadin fractions from cv. Keumkang flour. * indicates spot with ω-gliadin as a minor component. The epitopes for WDEIA in ω5-gliadin are marked such as QQIPQQQ (Yellow background), QQFPQQQ (Red background), QQSPEQQQ (Blue box) and QQSPQQQ (Black box).

## Slide 4
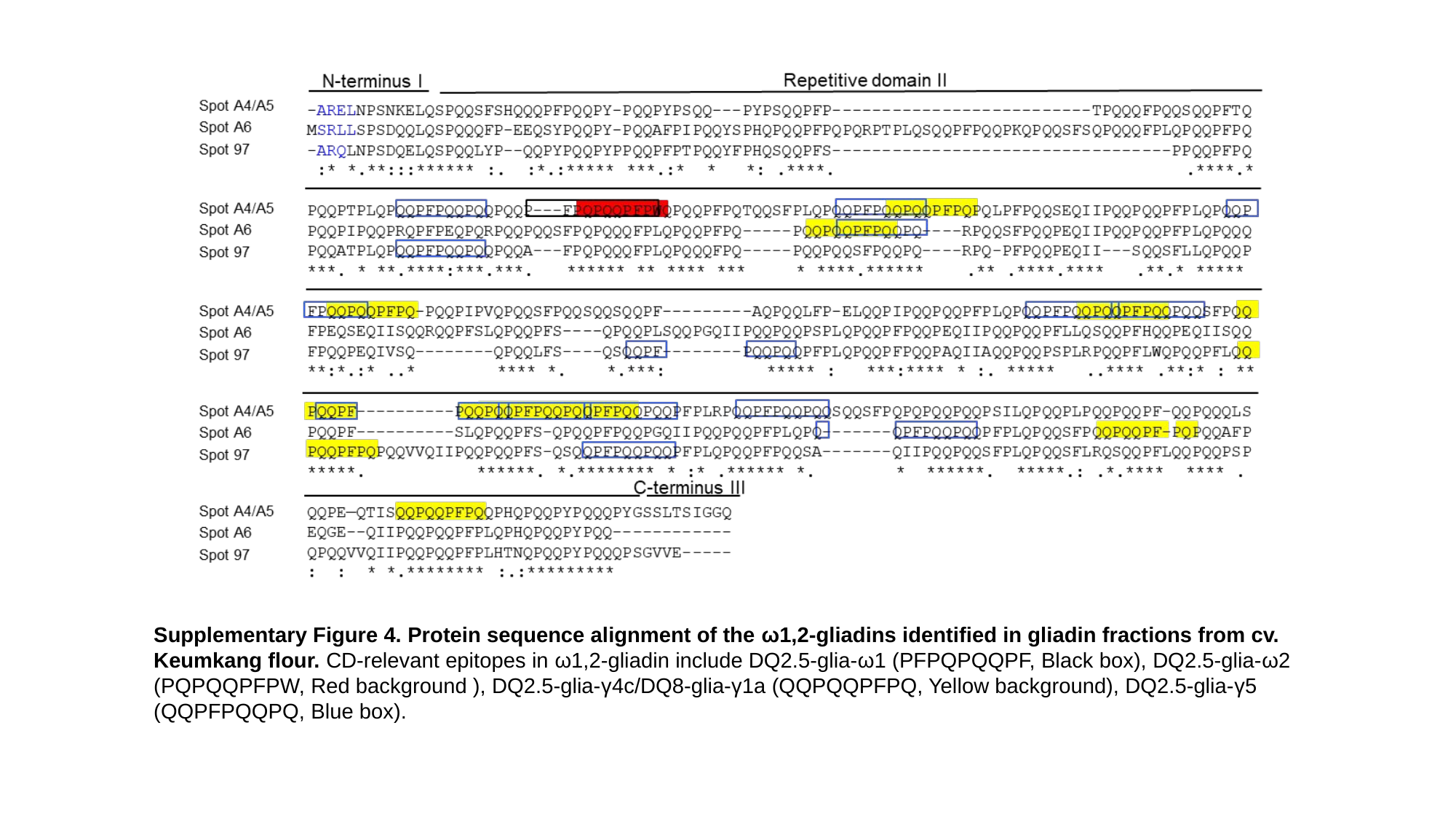

Supplementary Figure 4. Protein sequence alignment of the ω1,2-gliadins identified in gliadin fractions from cv. Keumkang flour. CD-relevant epitopes in ω1,2-gliadin include DQ2.5-glia-ω1 (PFPQPQQPF, Black box), DQ2.5-glia-ω2 (PQPQQPFPW, Red background ), DQ2.5-glia-γ4c/DQ8-glia-γ1a (QQPQQPFPQ, Yellow background), DQ2.5-glia-γ5 (QQPFPQQPQ, Blue box).

## Slide 5
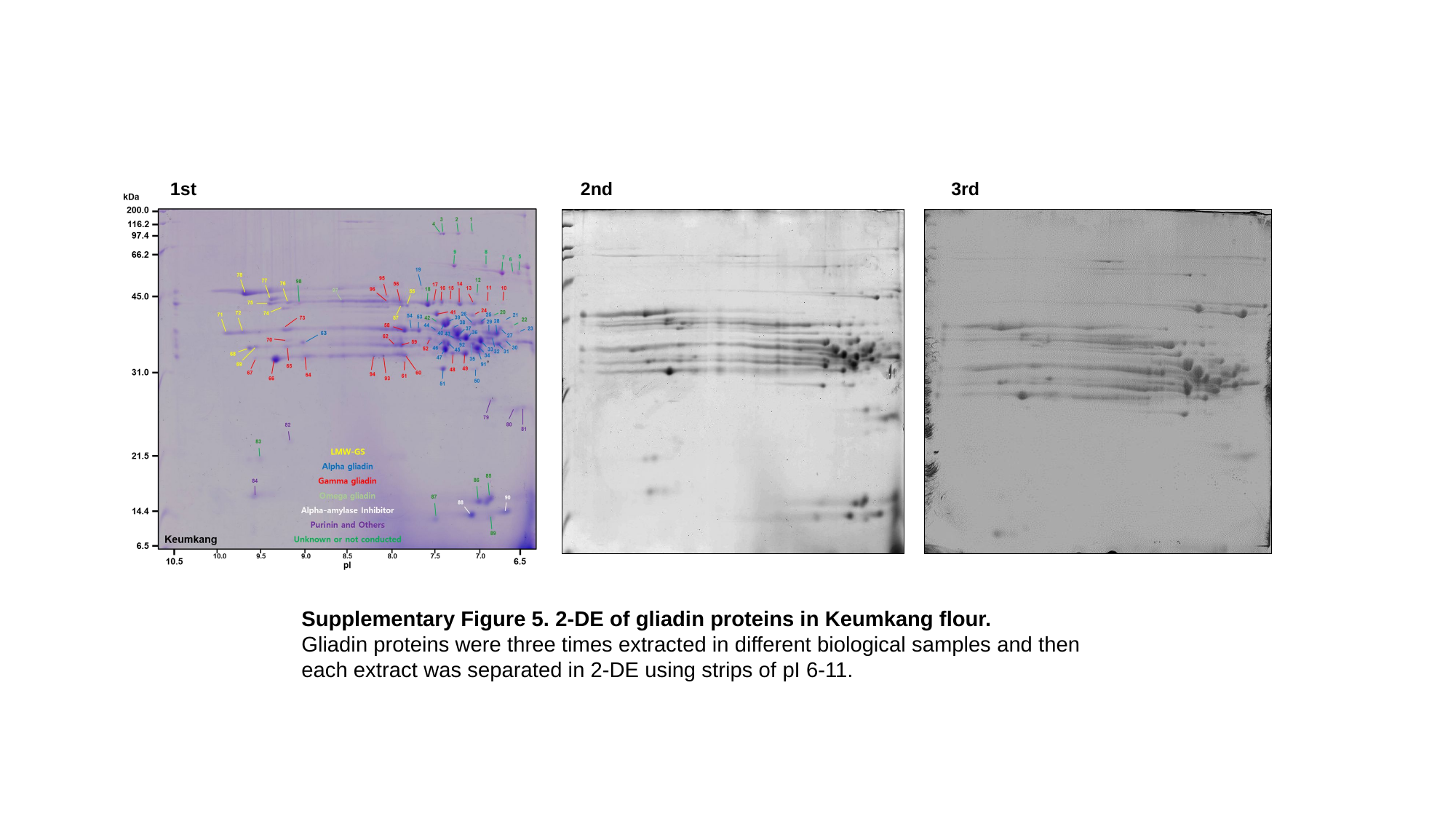

1st
2nd
3rd
Supplementary Figure 5. 2-DE of gliadin proteins in Keumkang flour.
Gliadin proteins were three times extracted in different biological samples and then each extract was separated in 2-DE using strips of pI 6-11.

## Slide 6
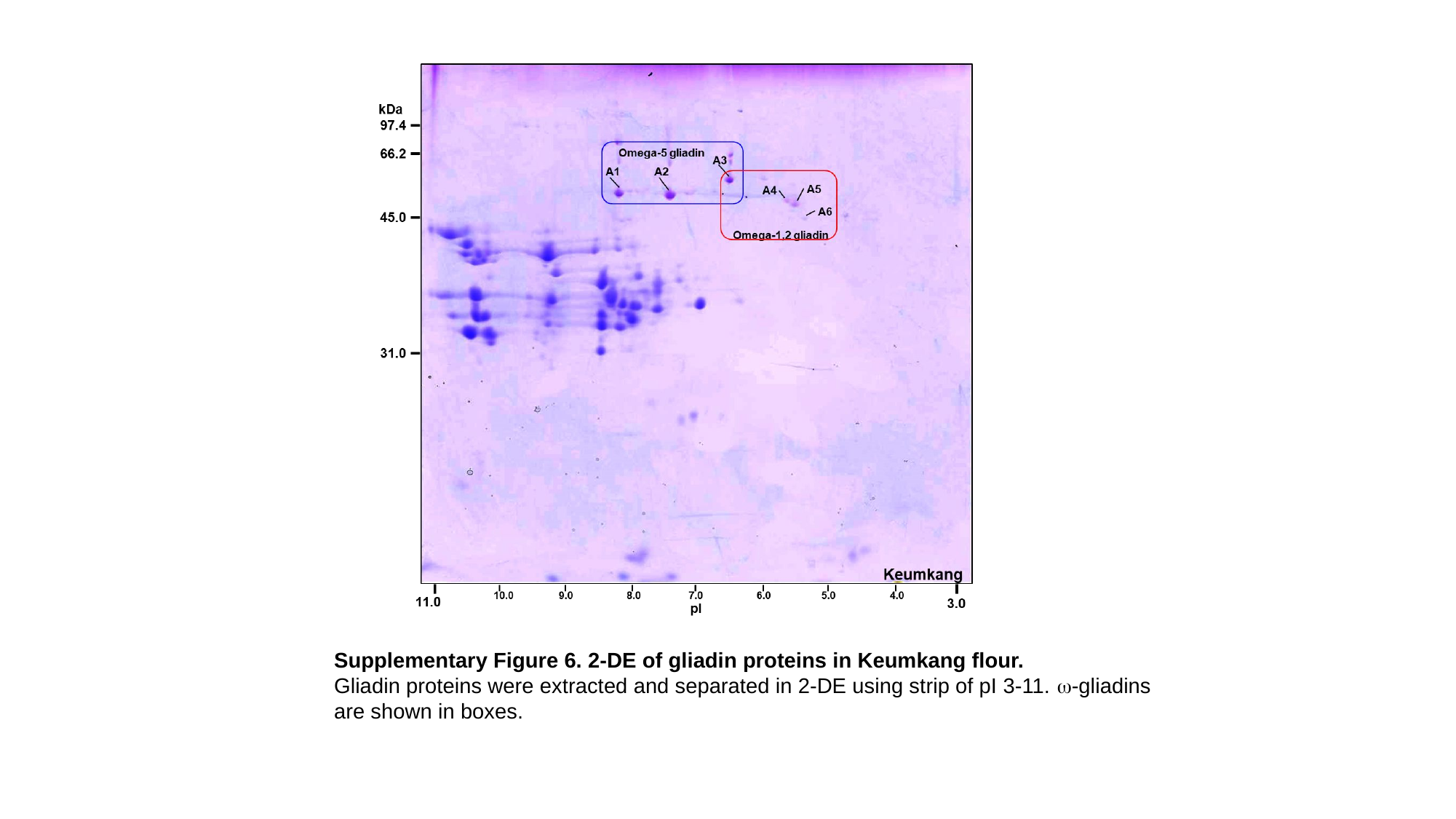

Supplementary Figure 6. 2-DE of gliadin proteins in Keumkang flour.
Gliadin proteins were extracted and separated in 2-DE using strip of pI 3-11. w-gliadins are shown in boxes.
